# Suppressed androgen receptor expression promotes M2 macrophage reprogramming through the STAT3/SOCS3 pathway

**Published:** 2019-01-24

**Authors:** Wenhan Ma, Jingbo Zhang, Linlin Guo, Ya Wang, Shuai Lu, ZhaoHui Wang, Qinghua Lu, Fengtao Wei

**Affiliations:** 1 Department of Internal Cardiology, the Second Hospital of Shandong University, Jinan, China; 2 Laboratory of Cardiovascular Immunology, Institute of Cardiology, Union Hospital, Tongji Medical College of Huazhong University of Science and Technology, Wuhan, China

**Keywords:** myocarditis, inflammation, androgen receptor, M2 macrophages, SOCS3, STAT3

## Abstract

Macrophages are important mediators of inflammatory cardiovascular diseases, and various macrophage phenotypes exert opposite effects during inflammation. In our previous study, we proved that suppressed androgen receptor (AR) alleviated inflammation during experimental autoimmune myocarditis (EAM). As anti-inflammatory cells, whether M2 macrophages are involved in this process remains unclear. Here, we showed that anti-inflammatory cytokines and M2 macrophages were elevated when AR was suppressed during EAM. In IL-4 stimulation-induced M2 macrophages, impaired AR with ASC-J9 increased the expression of M2 macrophage-related factors. Moreover, suppressed AR expression resulted in macrophage M2 polarization by reducing SOCS3 production and enhancing STAT3 activation. Taken together, our data suggest that AR plays a critical role in macrophage polarization and suppressed redundant AR expression promotes anti-inflammatory M2 macrophages reprogramming. This study suggests a potential therapeutic agent for inflammatory cardiomyopathy through the use of ASC-J9.

## Introduction

Myocarditis is characterized by inflammation of the cardiac muscle. The development of severe myocardial injury or the persistence of the immune response may lead to dilated cardiomyopathy or heart failure (Andreoletti et al., 2009[2]). Several lines of evidence have suggested that monocytes/macrophages represent the majority of inflammatory cells, thereby subserving a critical role in myocarditis (Afanasyeva et al., 2004[1]; Jaquenod De Giusti et al., 2015[11]; Leuschner et al., 2015[16]).

Macrophages display tremendous plasticity during their activation in response to various stimulators. At the two extreme ends of the spectrum of activation are two polarization states: the classical activation state (M1) and the alternative activation state (M2) (Gordon, 2003[8]). M1 macrophages are generated upon exposure to LPS and IFN-γ that release pro-inflammatory cytokines, such as TNF-α, IL-1β and IL-6. The enhanced expression of major histocompatibility complex class II (MHC-II) on M1 macrophages promotes the T cell activation involved in chronic inflammatory diseases (Boniakowski et al., 2017[4]). Conversely, in response to IL-4 and IL-13, macrophages are polarized to an immunosuppressive M2 state. M2 macrophages are characterized by the increased activity of argiynase-1 (Arg-1) and the surface expression of macrophage mannose receptor (MMR or CD206), secreting high levels of anti-inflammatory cytokines (IL-10) and playing a beneficial role in tissue remodeling and wound healing (Mohammadi et al., 2018[22]; Shapouri-Moghaddam et al., 2018[25]).

Studies have suggested that M1 macrophages are increased during myocarditis and that they promote inflammation in the development of myocarditis (Su et al., 2016[27]; Lu et al., 2018[18]). The adoptive transfer of *ex vivo*-programmed M1 macrophages significantly increased myocarditis (Li et al., 2009[17]). By contrast, polarization to M2 macrophages significantly restricted the inflammatory response of the myocardium in myocarditis (Zhang et al., 2016[33]; Wang et al., 2017[30]). The transfer of M2 macrophages remarkably alleviated myocardial inflammation, especially in men (Li et al., 2009[17]). Thus, it may be speculated that regulators of macrophage polarization may also exert pivotal functions in modulating heart inflammatory responses. 

The recently discovered androgen receptor is known to control inflammatory responses and regulate macrophage function (Lai et al., 2012[15]; Bizzaro et al., 2018[3]). In our previous study, we proved that the androgen receptor exacerbates inflammation in experimental autoimmune myocarditis, and the degradation enhancer of androgen receptor (ASC-J9®) alleviates inflammation by reducing M1 macrophage polarization (Ma et al., 2017[19]). Nevertheless, it remains unknown as to whether androgen receptor influences M2 polarization.

In this study, we discovered that ASC-J9 promoted M2 macrophage reprogramming both in myocarditis and *in vitro*. Reduced androgen receptor expression and activation in macrophages promoted M2-like polarization possibly via reduced SOCS3 expression. 

## Materials and Methods

### Animal experiments

Balb/c mice were injected with the murine a-MyHC sequence (MyHC-a614-629: acetyl SLKLMATLFSTYAS) emulsified 1:1 with complete Freund’s adjuvant (CFA, Sigma) on days 0 and 7. ASC-J9® was purchased from MedChem Express (Princeton, NJ/USA). The mice were intraperitoneally injected with ASC-J9® (75 mg/kg of body weight) every other day between days 8 and 20 of EAM. In parallel, the vehicle group received saline containing 0.5 % dimethyl sulfoxide (DMSO) (Sigma-Aldrich, Germany). All animals were treated according to the guidelines of the Guide for the Care and Use of Laboratory Animals published by the National Institutes of Health (NIH) and supported by the Animal Care and Utilization Committee of Huazhong University of Science and Technology.

### Immunofluorescence

The slides were incubated with primary antibodies against F4/80 (BD, Biosience, USA), Arg-1(Proteintech, USA) at 4 °C overnight. The slides were then incubated with secondary antibody for 1 h at 4 °C. Sections were analyzed using Image-Pro Plus software.

### Cell culture and treatment

The Raw264.7 cell line (provided by Pro. Zhang Mingxiang from Qilu Hospital of Shandong University) was cultured in DMEM (Sigma-Aldrich, Germany) medium supplemented with 10 % heat-inactivated fetal bovine serum (Sigma-Aldrich, Germany). A total of 1 x 10^5^ cells/well were seeded in 12-well plates and incubated overnight before treatment. After the cells were pretreated with ASC-J9 for 2 h, they were stimulated with IL-4 (20 ng/ml). 

### Quantitative RT-PCR

Total RNA was extracted using TRIzol® reagent (Takara Biotechnology, Japan) according to the manufacturer's protocol and converted into cDNA using a PrimeScript RT Reagent Kit (Takara Biotechnology, Japan). Relative mRNA expression was normalized to GAPDH mRNA levels.

### Western blot analysis

Cells were lysed in RIPA extraction solution containing protein inhibitor cocktail. Protein concentration was assessed using the BCA assay. Total protein lysates (60 µg/lane) were subjected to SDS-PAGE and transferred onto PVDF membranes (Millipore, USA.). The antibodies used were: GAPDH (1:1000, Antigene, China); SOCS3 (1:1000, CST, USA); STAT3 (1:1000, Immunoway, USA); and p-STAT3 (1:500, Immunoway, USA). HRP-labeled secondary antibody was used to identify the binding sites of the primary antibodies. Western blots were quantified by Quantity One Analysis software (Bio-Rad).

### Statistical analysis

Comparisons between groups were made using either the unpaired Student's t-test or the one-way ANOVA with GraphPad Prism 6.0 and SPSS 16.0 software. The data are presented as either the mean ± SEM or percentages. P-values < 0.05 were considered statistically significant. 

## Results

### ASC-J9-treated mice expressed increased levels of anti-inflammatory cytokines during EAM

**Figure 1:**
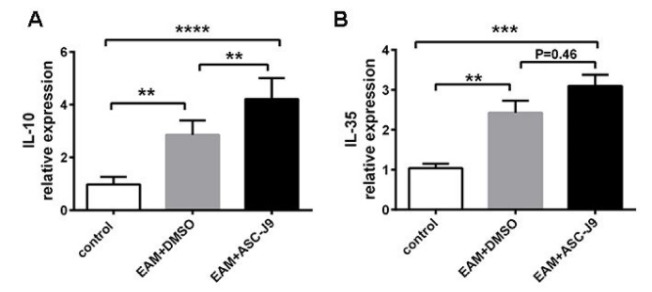
The expression of anti-inflammatory cytokines in ASC-J9-treated EAM mice. (A): The expression of IL-10 on mRNA levels in each group. (B): The expression of IL-35 on mRNA levels in each group. DMSO: saline containing 0.5 % DMSO. ASC-J9: an AR degradation enhancer *P < 0.05; **P < 0.01; ***P < 0.005; ****P < 0.001

In our previous study, we proved that AR was elevated in EAM. ASC-J9 alleviated inflammation and myocardial injury by reducing inflammatory cytokines and M1 macrophage during EAM (Ma et al., 2017[19]). To analyze changes of anti-inflammatory cytokines during ASC-J9 administration, we first measured the expression levels of IL-10 in myocardium. As Figure 1A(Fig. 1) shows that IL-10 levels were elevated in ASC-J9-treated EAM mice. Treg cells serve as suppressors of excessive immune responses via the production of immunosuppressive cytokines, including IL-35, which play a protective role during EAM (Yan et al., 2016[32]). Next, we measured the levels of IL-35 during ASC-J9 administration. As we expected, the expression levels of IL-35 were higher in the ASC-J9 group than in the DMSO group (Figure 1B(Fig. 1)). These results indicated that the suppression of AR by ASC-J9 treatment promoted the expression of anti-inflammatory cytokines during EAM.

### Suppressed AR expression upregulated M2 macrophages in EAM

**Figure 2:**
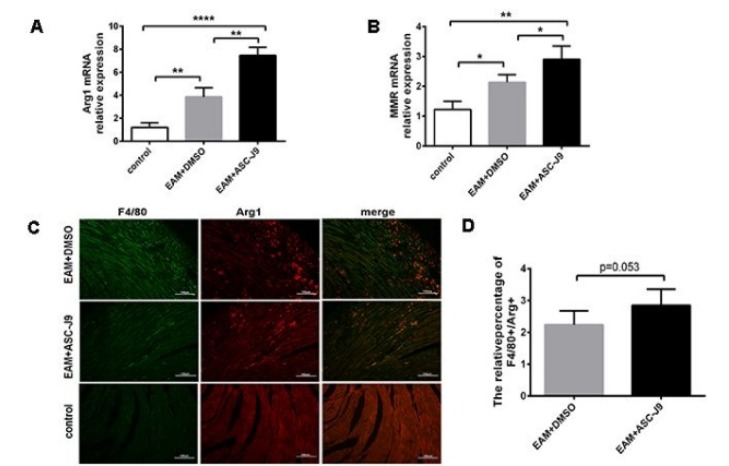
Suppressed AR expression promotes M2 macrophages infiltration in EAM mice. (A, B): mRNA expression levels of Arg1 and MMR in the myocardium of each group. (C, D): Immunofluorescence images of Arg1 and F4/80 double-positive macrophages and the analysis of the relative proportion of double-positive cells in each group (n=10). *P < 0.05; **P < 0.01; ***P < 0.005; ****P < 0.001

Several lines of data have indicated that macrophages play an indispensable role in the development of myocarditis, and M2 macrophages display a protective role in myocarditis (Zhou et al., 2018[35]). Given that our data suggested that ASC-J9 decreased inflammation in EAM, we speculated that M2 macrophages may be distinct in the ASC-J9 and vehicle groups. We examined the phenotype of M2 macrophages in ASC-J9- and DMSO-treated mouse heart infiltrates using immunofluorescence. We found that the minimal level of F4/80^+^Arg-1^+^ double-positive macrophages were detected in the EAM group. After ASC-J9 treatment, the relative percentage of F4/80^+^Arg-1^+^ double-positive macrophages was slightly higher (Figure 2C, D(Fig. 2)). To further investigate the changes of M2 macrophage in ASC-J9-treated mice, mononuclear cells were derived from the myocardium of each group. The expression of M2-specific gene Arg1 and the specific surface marker MMR were significantly elevated in the ASC-J9 group (Figure 2A, B(Fig. 2)). Taken together, these data indicated that AR had substantial influence over M2 macrophage polarization during EAM.

### ASC-J9 facilitated M2 macrophage polarization in vitro

**Figure 3:**
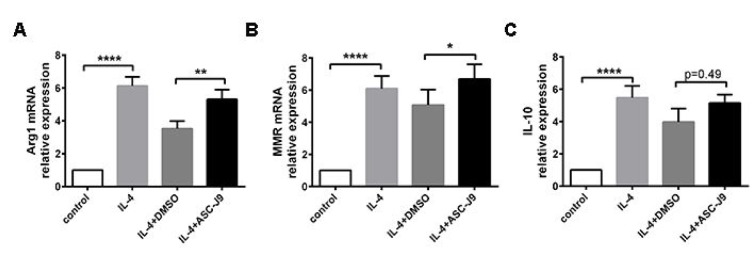
Suppressed AR expression promotes Arg1 (A), MMR (B) and IL-10 (C) expression in Raw264.7 cells within IL-4 stimulation (n=6). *P < 0.05; **P < 0.01; ***P < 0.005; ****P < 0.001

Macrophage polarization needs to properly receive and coordinate signals provided by specific inflammatory cytokines that mediate their development. To determine whether impaired AR participates in macrophage polarization to M2 phenotype, RAW264.7 cells were prepared. In M2 macrophage polarization, ASC-J9, an enhancer of degradation of AR, was added to Raw264.7 culture followed by 20 ng/ml IL-4 treatment for 48 h. The expression levels of M2 macrophage-related molecules, including MMR, Arg1 and IL-10 were significantly higher after IL-4 treatment, and ASC-J9 stimulation further upregulated MMR, IL-10 and Arg1 compared with IL-4-treated macrophages (Figure 3A, B, C(Fig. 3)).

### Suppressed AR expression promotes M2 macrophage polarization through SOCS3/STAT3 pathway

**Figure 4:**
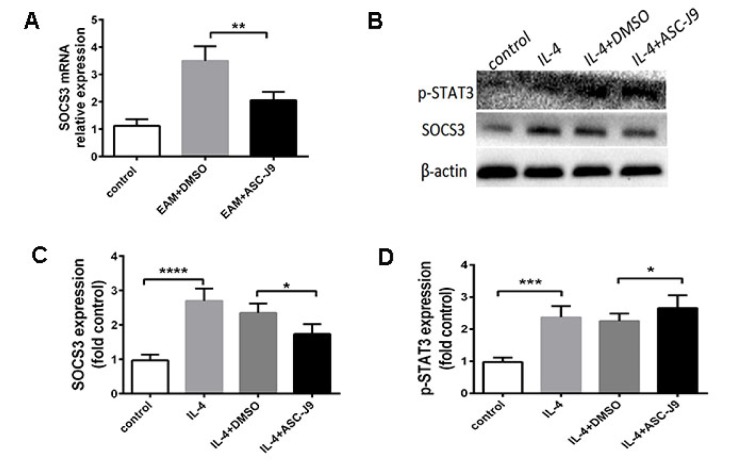
Suppressed AR reduced SOCS3 and enhanced STAT3 activation in vivo. (A): The expression of SOCS3 in ASC-J9-treated mice on the mRNA level. (B): Western blot image of SOCS3 and STAT3 phosphorylation. (C): The analysis of SOCS3 in each group (n=6). (D): The analysis of STAT3 phosphorylation in each group (n=6). *P < 0.05; **P < 0.01; ***P < 0.005; ****P < 0.001

We next sought to examine the molecular mechanisms by which AR suppression increased macrophage M2 polarization. It has been shown that suppressors of cytokine signaling (SOCS) family molecules serve as molecular switches that control immune activation/suppression and M1/M2 macrophage polarization (Wilson, 2014[31]). In ASC-J9-treated mice, we observed decreased expression of SOCS3 in mRNA levels (Figure 4A(Fig. 4)). To confirm our results *in vitro*, we treated Raw264.7 cells with IL-4. As the results reflected, macrophages in the ASC-J9-pretreated group exhibited significantly lower levels of SOCS3 than did vehicle group macrophages (Figure 4B, C). The JAK/STAT3/SOCS3 signaling pathway has been reported to exert a crucial role in M2 macrophages (Tang et al., 2017[29]). To investigate whether STAT3 was involved in the enhanced M2 polarization of ASC-J9-treated macrophages, we measured STAT3 activation in each group. We found that ASC-J9 administration enhanced STAT3 phosphorylation (Figure 4D(Fig. 4)). These data suggested that STAT3 may involve AR-mediated macrophage M2 polarization. 

## Discussion

AR has been recognized as an important regulator of androgen-mediated signaling. It has recently become appreciated that AR is linked to innate and adaptive immunity (Lai et al., 2012[15]), especially in macrophage-mediated inflammation (Huang et al., 2015[9]; Morooka et al., 2016[23]; Bizzaro et al., 2018[3]). Chang reported that inhibiting AR expression in macrophages reduced inflammation by impaired macrophage migration and inflammation cytokines secretion both in atherosclerosis and wound healing (Lai et al., 2009[14]; Huang et al., 2014[10]). The resolution of cardiac inflammation and injury is a therapeutic pathway for myocarditis and subsequent dilated cardiomyopathy (Medina et al., 2017[21]; Sun et al., 2018[28]). Our previous study showed that suppressing AR by ASC-J9 attenuated cardiac inflammation and injury by reducing M1 macrophages (Ma et al., 2017[19]). We further demonstrated that suppressed AR expression increased anti-inflammatory cytokines and M2 macrophages during EAM.

Myocarditis is an inflammatory disease that has been attributed to Th17/Treg imbalance (Yan et al., 2016[32]). Nevertheless, as important immune cells, macrophages play a crucial role in myocarditis; in particular, differential-phenotype macrophages may generate an opposite inflammatory response (Jaquenod De Giusti et al., 2015[11]). Several lines of evidence have suggested that the promotion of M2 macrophage polarization plays a protective role in myocarditis (Wang et al., 2017[30]; Karuppagounder et al., 2018[12]; Kovacevic et al., 2018[13]). In our study, we found significantly increased expression of M2 macrophages in mice when AR was suppressed, despite the fact that the relative ratio of M2 macrophages on fluorescence staining was only slightly increased with no statistical significance. This may be due to the statistical inaccuracy of fluorescence staining. To further investigate whether AR inhibition regulated M2 macrophage polarization *in vitro*, we analyzed the M2 marker in ASC-J9 pretreated Raw264.7 cells. IL-4 is produced by various innate cells, including non-B non-T cells, mast cells, NKT cells and even macrophages themselves (Gordon, 2003[8]) and is a potent stimulus of M2 macrophage polarization. In the present study, we found that impairing AR with ASC-J9 promoted Arg1, MMR and IL-10, all of which are specific markers of M2 macrophages under IL-4 stimulation. Therefore, impaired AR expression ameliorated cardiac inflammation through sharply suppressed M1-like and promoted M2-like macrophage phenotype.

Profound new discoveries have shown that the disparate SOCS family molecules may serve as molecular switches that control immune activation/suppression and M1/M2 macrophage polarization (Zhou et al., 2017[34]). In IL-4-stimulated M2 macrophage polarization, a study proved that activating the JAK1/STAT3/SOCS3 signaling pathway contributed to M2 macrophage reprogramming in acute lung injury (Fang et al., 2017[7]). They confirmed that proper M2 polarization was associated with enhanced STAT3 phosphorylation and decreased SOCS3 expression (Tang et al., 2017[29]). Moreover, impaired SOCS3 feedback led to permissive IL10/STAT3 signaling that promoted M2 macrophage activation (Nakamura et al., 2015[24]). Since suppressed AR expression promotes M2 macrophage and increased IL-10 expression *in vivo* and *in vitro*, we speculated that the STAT3/SOCS3 signaling pathway was involved in this process. Consistent with these previous studies, we observed that SOCS3 was decreased both in the hearts of ASC-J9-treated mice and in Raw264.7 cells under Il-4 stimulation. We also showed that IL-4 induced a dramatic elevation of STAT3 phosphorylation in the ASC-J9-treated group. Taken together, our data suggested that suppressed AR expression regulates macrophage M2 polarization possibly through the SOCS3-STAT3 signaling axis.

AR is a member of the nuclear receptor (NR) gene superfamily and acts as a ligand-dependent transcription factor (Matsumoto et al., 2013[20]). It has been reported that AR regulates CCR-2 and TNF-a expression by binding to the androgen-response-element (ARE) of these genes. Therefore, we speculate that AR may regulate SOCS3 expression through genomic pathway. To verify our hypothesis, we found that there were predicted AREs on the promoter region of SOCS3 using the ALGGEN PROMO system. In addition to the classical paradigm in which AR exerts its biological effects in the nucleus by orchestrating the expression of the androgen-regulated transcriptome, there is considerable evidence supporting nongenomic activity of AR (Simoncini and Genazzani, 2003[26]). Other studies have shown that AR exerts its function rapidly by regulating the AKT signaling pathway (Cinar et al., 2007[5]; Deng et al., 2017[6]). Whether AR regulates M2 macrophages polarization through a genomic or nongenomic pathway warrants further investigation. 

## Conclusion

Taken together, these findings indicated that suppressed AR promotes anti-inflammatory cytokine expression and facilitates M2 macrophage polarization through the STAT3/ SOCS3 pathway. Therefore, suppressed AR expression in macrophages may be a therapeutic method in myocarditis, especially in males. ASC-J9 may be thought of as a complementary therapeutic agent in the protection against cardiac damage in inflammatory cardiomyopathy.

## Notes

Wenhan Ma and Jingbo Zhang contributed equally to this work.

## Acknowledgements

This work was supported by a grant from the Natural Science Foundation of Shandong Provence, China (ZR2018PH004, BS2011YY011), and the Youth Foundation of Second Hospital, Shandong University, Jinan, China (2018YT05, Y2013010023)

## Conflict of interest

The authors declare that they have no conflict of interest.
